# Development and validation of a nomogram for preoperatively predicting permanent stoma after rectal cancer surgery with ileostomy: a retrospective cohort study

**DOI:** 10.1186/s12885-024-12642-7

**Published:** 2024-07-22

**Authors:** Chenglin Tang, Fan He, Fuyu Yang, Defei Chen, Junjie Xiong, Yu Zou, Kun Qian

**Affiliations:** Department of Gastrointestinal Surgery, The First Affiliated Hospital of Chongqing Medical University, Chongqing, 400016 China

**Keywords:** Temporary ileostomy, Permanent stoma, Rectal cancer, Preoperative risk factor, Nomogram

## Abstract

**Background:**

For patients with rectal cancer, the utilization of temporary ileostomy (TI) has proven effective in minimizing the occurrence of severe complications post-surgery, such as anastomotic leaks; however, some patients are unable to reverse in time or even develop a permanent stoma (PS). We aimed to determine the preoperative predictors associated with TS failure and develop and validate appropriate predictive models to improve patients’ quality of life.

**Methods:**

This research included 403 patients with rectal cancer who underwent temporary ileostomies between January 2017 and December 2021. All patients were randomly divided into either the developmental (70%) or validation (30%) group. The independent risk factors for PS were determined using univariate and multivariate logistic regression analyses. Subsequently, a nomogram was constructed, and the prediction probability was estimated by calculating the area under the curve (AUC) using receiver operating characteristic (ROC) analysis. A calibration plot was used to evaluate the nomogram calibration.

**Results:**

Of the 403 enrolled patients, 282 were randomized into the developmental group, 121 into the validation group, and 58 (14.39%) had a PS. The development group consisted of 282 patients, of whom 39 (13.81%) had a PS. The validation group consisted of 121 patients, of whom, 19 (15.70%) had a PS; 37 related factors were analyzed in the study. Multivariate logistic regression analysis demonstrated significant associations between the occurrence of PS and various factors in this patient cohort, including tumor location (OR = 6.631, *P* = 0.005), tumor markers (OR = 2.309*, P* = 0.035), American Society of Anesthesiologists (ASA) score (OR = 4.784*, P* = 0.004), T4 stage (OR = 2.880*, P* = 0.036), lymph node metastasis (OR = 4.566*, P* = 0.001), and distant metastasis (OR = 4.478, *P* = 0.036). Furthermore, a preoperative nomogram was constructed based on these data and subsequently validated in an independent validation group.

**Conclusion:**

We identified six independent preoperative risk factors associated with PS following rectal cancer resection and developed a validated nomogram with an area under the ROC curve of 0.7758, which can assist surgeons in formulating better surgical options, such as colostomy, for patients at high risk of PS.

## Background

Colorectal cancer ranks as the third most prevalent malignancy globally [1]. Due to advancements in the quality of modern life and the impact of the surrounding environment, coupled with increased awareness and affordability of colorectal cancer screening, as well as enhanced screening capacity, there has been a significant surge in the incidence of colorectal cancer [2]. The increase in patient incidence and the early detection of tumors have resulted in a corresponding surge in surgical procedures. Nevertheless, surgeons inevitably encounter decisions regarding the choice of surgical method and perioperative complications during surgical treatment. To mitigate the occurrence of complications such as anastomotic fistula, temporary stoma procedures such as ileostomy or colostomy are implemented alongside anterior resection for patients diagnosed with low rectal cancer [3]. Anastomotic leakage in the rectum is a significant complication, with a mortality rate ranging from 2% to 16.4%, highlighting the importance of temporary stoma (TS) in rectal anastomosis [4]. After low anterior resection for rectal cancer, a diverting loop stoma is recommended to reduce the incidence of anastomotic leakage [5, 6]. Following surgery for low rectal cancer, an ostomy is typically necessary to mitigate potential complications, and most physicians opt for an ileostomy due to its advantages, including ease of reduction. To facilitate subsequent surgical procedures, temporary ileostomy is commonly opted for by most physicians. However, the subgroup of patients who are unable to undergo a second surgery for various reasons are often neglected. Moreover, studies have shown that up to 30% of patients with ostomies develop ostomy-related complications, which leads to a decline in their overall well-being and imposes significant financial strain on the entire household [7, 8]. The overall incidence of complications associated with stomas, including skin irritation, high-output ostomy, outlet obstruction, stoma prolapse, parastomal hernia, dehydration, and reduced renal function, was significantly higher in ileostomies than in colostomies [9, 10].

Although temporary ostomy is performed on certain patients, restoration may not be feasible due to various factors, resulting in persistent complications associated with permanent ostomies when reversal procedures fail [11]. In clinical practice, the overlooked risk factors for the conversion of a temporary stoma (TS) to a permanent stoma (PS) prompted us to investigate whether a preoperative model capable of assessing the likelihood of restoration failure exists for high-risk patients with ileostomy difficulty. Such a model would facilitate improved patient communication regarding their condition and treatment plans, improve postoperative long-term quality of life for patients, enhance patient satisfaction, and reduce complications. Therefore, timely preoperative detection of high-risk patients who cannot undergo temporary ileostomy (TI) reversal is conducive to preoperative consultation and surgical planning. For this subset of patients with a potentially permanent stoma, colostomy or Hartmann surgery may be more beneficial for long-term prognosis. The objective of this study was to determine the preoperative predictors associated with TS failure and to develop and validate appropriate predictive models that could improve patients’ quality of life while reducing their financial burden and mental stress.

## Methods

### Patients

After obtaining approval from the Institutional Review Board, we conducted a retrospective analysis of the clinical data of patients who underwent rectal cancer surgery combined with ileostomy at the First Affiliated Hospital of Chongqing Medical University between January 2017 and December 2021. The inclusion criteria for patients in the study were as follows: 1) pathologically confirmed rectal cancer and 2) the surgical procedure was radical resection of rectal cancer combined with ileostomy at our hospital. Exclusion criteria were 1) Incomplete clinical data and 2) Loss to follow-up. A total of 403 patients were included in the study, following the exclusion of 37 out of 440 patients. Of the 37 patients excluded, 35 were lost to follow-up, and 2 lacked imaging and pathology data. There were 345 patients in the TS group and 58 patients in the PS group. In previous studies, the median time from the initial surgery to stoma closure was 6.9 months, and our findings also indicated that a minimal proportion of patients underwent stoma closure beyond one year. Therefore, in this study, we defined PS as a temporary stoma that had not been reversed within one year.

### Data collection

We retrospectively collected preoperative baseline and partial postoperative data. Baseline data included gender; age; preoperative comorbidities; previous abdominal surgery; height; weight; smoking history; alcohol consumption; hemoglobin, albumin, and fibrinogen levels; white blood cell count; preoperative neoadjuvant chemoradiotherapy; age-adjusted Charlson Comorbidity Index (ACCI); and tumor-related data, including the distance from the tumor to the anal margin and tumor markers. The Charlson Comorbidity Index (CCI) score was determined by tallying the presence of various comorbidities, such as myocardial infarction, congestive heart failure, peripheral vascular disease, dementia, cerebrovascular disease, rheumatoid disease, peptic ulcer disease, diabetes (with or without complications), chronic pulmonary disease, mild/moderate/severe liver disease, hemiplegia, moderate/severe renal disease, solid tumor (with or without metastasis), leukemia, lymphoma, and acquired immunodeficiency syndrome (AIDS). The ACCI score was then calculated based on this information by assigning 1 point for every 10 years of age for patients older than 40 years (0 points for those aged ≤ 40 years; 1 point for those aged 41–50; 2 points for those aged 51–60; 3 points for those aged 61–70; and 4 points for those aged > 70). The tumor markers examined in this study included alpha-fetoprotein (AFP), carcinoembryonic antigen (CEA), carbohydrate antigen 19–9, cytokeratin 19, and pro-gastrin releasing peptide. An abnormal result was defined as any marker exceeding the established normal range. Two experienced clinicians independently determined tumor staging by preoperative computed tomography enhancement, pelvic magnetic resonance imaging enhancement, and transrectal ultrasound, and further discussions were initiated in response to divergent opinions regarding the tumor stage of the patient. Postoperative data included tumor differentiation and pathological stage.

### Statistical analysis

R studio and SPSS were used for data processing and statistical analyses. The means ± SDs were used to report normally distributed continuous variables, medians (ranges) were used for skewed variables, and frequencies (percentages) were used for categorical variables. The optimal cut-off points for continuous independent variables were determined based on the highest Youden index (sensitivity + specificity - 1). Age was converted to a binary variable using a cutoff of 60 years, while the ACCI and distance from the tumor to the anal margin were converted to 5 points and 6 cm, respectively.

Continuous variables conforming to a Gaussian distribution are presented as the mean ± standard deviation (SD) and were compared using the t test. Categorical variables are expressed as counts and percentages, and chi-squared or Fisher's exact tests were used for comparisons. The patients were randomly allocated into development and validation groups in a 7:3 ratio using the R software set.seed function. The seed number was “1.” Univariate logistic regression analysis was conducted on all variables in the development group using SPSS. Only preoperatively relevant variables with a significance level of *P* < 0.05 in the univariate analysis were included in the multivariate logistic analysis. The findings are presented as odds ratios (ORs), 95% confidence intervals (CIs), and corresponding *P* values. Finally, the independent risk factors derived from the multivariate logistic analysis were further analyzed and plotted using R software. The rms package of R software was used to construct the nomograms, and the pROC package was used to construct the receiver operating characteristic (ROC) curves. Model calibration was evaluated using ROC curve goodness-of-fit tests. The model's calibration was evaluated by conducting goodness-of-fit tests on the ROC curve. Statistical significance was set at *P* < 0.05.

## Results

**Fig. 1:**
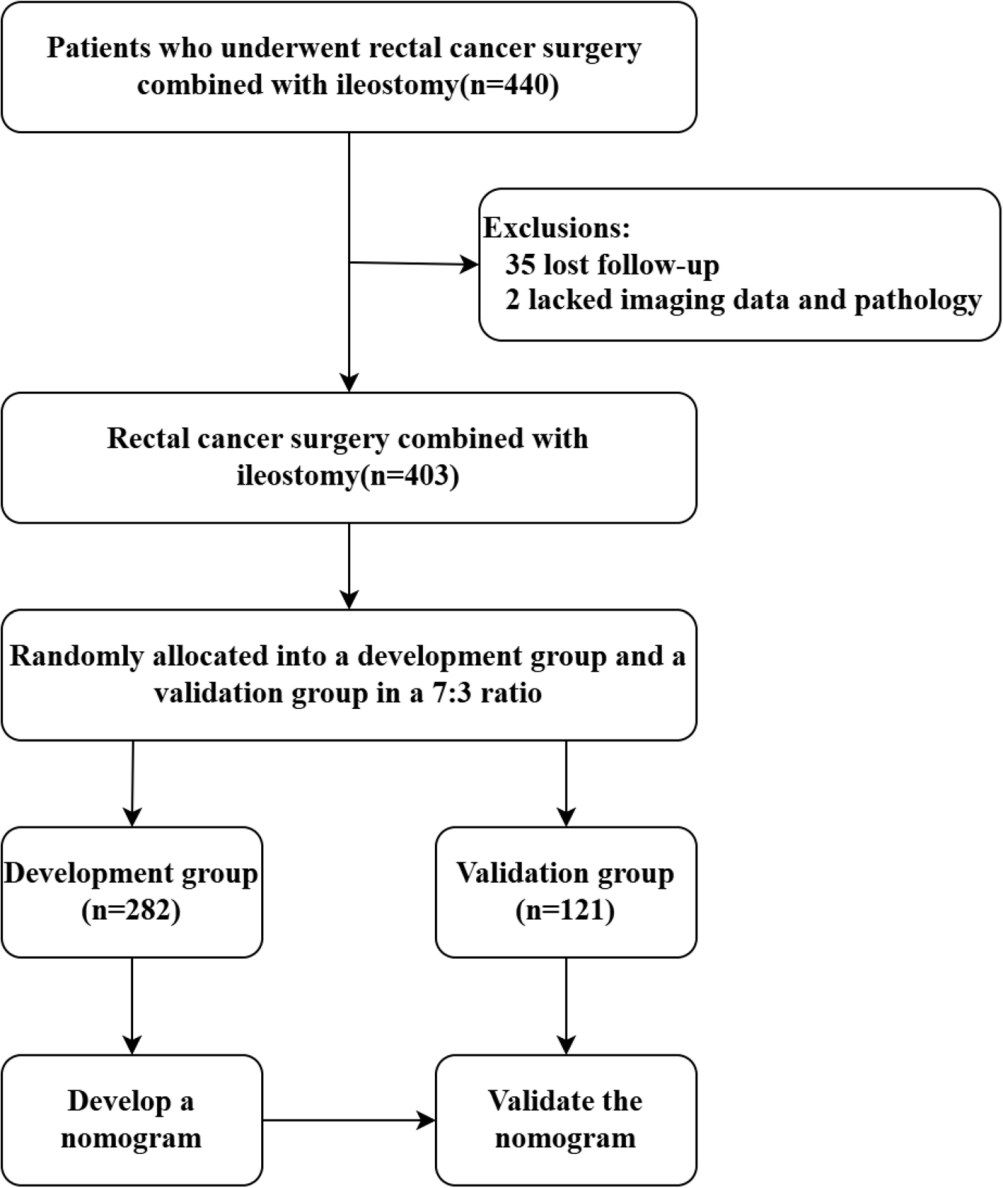
Flow chart of the study

Overall, 440 patients treated in our hospital from January 2017 to December 2021 were included in this study; 37 of these patients were excluded due to incomplete clinical data and loss to follow-up (Fig. 1). Among the 403 enrolled patients, 58 had PS.

Patients were randomly divided into development (282) and validation (121) groups, and univariate analysis revealed no significant difference between the two groups (Table 1). Among all 403 patients, the male population constituted the overwhelming majority, accounting for 67%. This observation may be attributed to the narrower pelvic inlet in males and the consequent surgical challenges. However, gender differences did not emerge as a significant factor in PS within the developmental group.

**Table 1:** Baseline information between the validation and development cohorts

Characteristics	Total (*n* = 403)	Validation (*n* = 121)	Development (*n* = 282)	*P* value
Permanent stoma, n (%)				0.737
No	345 (86)	102 (84)	243 (86)	
Yes	58 (14)	19 (16)	39 (14)	
Gender, n (%)				0.137
Female	133 (33)	33 (27)	100 (35)	
Male	270 (67)	88 (73)	182 (65)	
Age, year n (%)				0.919
≤ 60	160 (40)	49 (40)	111 (39)	
> 60	243 (60)	72 (60)	171 (61)	
Hypertension, n (%)	96 (24)	30 (25)	66 (23)	0.863
Diabetes, n (%)	41 (10)	13 (11)	28 (10)	0.946
Previous abdominal surgery, n(%)	87 (22)	26 (21)	61 (22)	1
Weight, kg Median (IQR)	60 (55, 68)	60 (55, 66)	60 (55, 68)	0.516
Height, cm Median (IQR)	163 (158, 168)	163(158, 168)	162 (158, 168)	0.594
BMI, Mean ± SD	23.13 ± 3.04	22.9 ± 3.24	23.23 ± 2.95	0.345
Smoking history, n (%)	180 (45)	55 (45)	125 (44)	0.921
Alcohol consumption, n (%)	139 (34)	41 (34)	98 (35)	0.957
Neoadjuvant chemotherapy, n (%)	112 (28)	29 (24)	83 (29)	0.317
Neoadjuvant radiotherapy, n (%)	81 (20)	20 (17)	61 (22)	0.300
Lung diseases, n (%)	132 (33)	36 (30)	96 (34)	0.468
Neuro diseases, n (%)	17 (4)	7 (6)	10 (4)	0.450
Heart diseases, n (%)	44 (11)	15 (12)	29 (10)	0.653
Other pre-complication, n (%)	68 (17)	19 (16)	49 (17)	0.790
Distance from tumor to anal margin, n (%)				1
> 6 cm	112 (28)	34 (28)	78 (28)	
≤ 6 cm	291 (72)	87 (72)	204 (72)	
Neutrophil, 10^9/L Median (IQR)	3.12 (2.45, 3.84)	3.17(2.45, 3.82)	3.1 (2.44, 3.85)	0.849
Hemoglobin, g/L Median (IQR)	130 (116.5, 140.5)	132 (120, 144)	128 (115, 140)	0.096
Lymphocyte, 10^9/L Median (IQR)	1.42 (1, 1.79)	1.42 (1.09, 1.75)	1.42 (0.96, 1.8)	0.976
Albumin, g/L Median (IQR)	41 (38, 44)	41 (38, 44)	41 (37, 44)	0.681
Fibrinogen, g/L Median (IQR)	3.27 (2.8, 3.77)	3.29 (2.77, 3.79)	3.26 (2.8, 3.76)	0.990
PNI, Mean ± SD	47.81 ± 5.6	47.82 ± 5.12	47.81 ± 5.81	0.983
NLR, Median (IQR)	2.32 (1.67, 3.24)	2.25 (1.78, 2.93)	2.34 (1.65, 3.26)	0.634
Tumor marker, n (%)				0.199
Normal	249 (62)	81 (67)	168 (60)	
Abnormal	154 (38)	40 (33)	114 (40)	
CRM, n (%)	40 (10)	12 (10)	28 (10)	1
EMVI, n (%)	52 (13)	10 (8)	42 (15)	0.097
ACCI, n (%)				0.211
≤ 5	209 (52)	69 (57)	140 (50)	
> 5	194 (48)	52 (43)	142 (50)	
ASA score, n (%)				0.944
< 3	354 (88)	107 (88)	247 (88)	
≥ 3	49 (12)	14 (12)	35 (12)	
Distance from anastomosis to anal margin, cm	3 (2, 4)	3 (2, 4)	3 (2, 4)	0.413
Median (IQR)				
T3 stage, n (%)	219 (54)	66 (55)	153 (54)	1
T4 stage, n (%)	46 (11)	15 (12)	31 (11)	0.814
LNM, n (%)	207 (51)	60 (50)	147 (52)	0.720
Distant metastasis, n (%)	25 (6)	6 (5)	19 (7)	0.650
Tumor type, n (%)				0.233
High differentiation	2 (0)	0 (0)	2 (1)	
Moderate differentiation	283 (70)	87 (72)	196 (70)	
Low differentiation	71 (18)	25 (21)	46 (16)	
other	47 (12)	9 (7)	38 (13)	
Pathological stage, n (%)				0.473
0	17 (4)	4 (3)	13 (5)	
1	129 (32)	37 (31)	92 (33)	
2	113 (28)	41 (34)	72 (26)	
3	125 (31)	35 (29)	90 (32)	
4	19 (5)	4 (3)	15 (5)	

*IQR* Interquartile range, *BMI* Body mass index, *PNI* Prognostic nutritional index, *NLR* Neutrophil-to-Lymphocyte Ratio, *CRM* Circumferential resection margin, *EMVI*, Extramural venous invasion, *ACCI* Age-adjusted Charlson Comorbidity Index, *ASA* American Society of Anesthesiologists, *LNM* lymph node metastasis

Univariate analysis of the developmental group data revealed significant differences in the following variables: age (*P* = 0.012), tumor distance (*P* = 0.007), abnormal tumor markers (*P* = 0.005), ACCI (*P* = 0.005), ASA score* (P* < 0.001), T4 stage (P = 0.012), lymph node metastasis (*P* = 0.001), distant metastasis (*P* = 0.027), and postoperative pathological stage(*P* < 0.001) (Table 2).

**Table 2:** Univariate logistic regression analysis in development group

Characteristics		OR	95%CI	P Value
Gender	(male/female)	1.706	0.794–3.664	0.171
Age	(> 60/ ≤ 60)	2.851	1.258–6.459	0.012
Hypertension	(yes/no)	0.979	0.439–2.183	0.959
Diabetes	(yes/no)	1.407	0.501–3.949	0.517
Previous abdominal surgery	(yes/no)	1.298	0.594–2.838	0.513
Weight	(by 1 kg)	1.015	0.981–1.051	0.384
Height	(by 1 cm)	1.014	0.972–1.058	0.518
BMI	(by 1)	1.039	0.926–1.165	0.517
Smoking history	(yes/no)	0.666	0.330–1.343	0.256
Alcohol consumption	(yes/no)	1.060	0.523–2.147	0.871
Neoadjuvant chemotherapy	(yes/no)	0.685	0.310–1.515	0.350
Neoadjuvant radiotherapy	(yes/no)	0.925	0.401–2.132	0.855
Lung disease	(yes/no)	1.604	0.807–3.190	0.178
Neuro disease	(yes/no)	1.588	0.325–7.769	0.568
Heart disease	(yes/no)	0.995	0.327–3.037	0.995
Other precomplication	(yes/no)	0.846	0.334–2.144	0.724
Distance from tumor to anal margin	(> 6 cm/ ≤ 6 cm)	5.357	1.599–17.944	0.007
Neutrophil	(by 10^9/L)	0.959	0.751–1.225	0.737
Hemoglobin	(by 1 g/L)	0.992	0.947–1.009	0.347
Lymphocyte	(by 10^9/L)	1.054	0.615–1.806	0.849
Albumin	(by 1 g/L)	1.000	0.929–1.077	0.992
Fibrinogen	(by 1 g/L)	1.229	0.778–1.942	0.376
PNI	(by 1)	1.003	0.946–1.064	0.912
NLR	(by 1)	0.994	0.826–1.196	0.946
Tumor marker	(abnormal/normal)	2.720	1.357–5.454	0.005
CRM	(yes/no)	1.043	0.341–3.187	0.941
EMVI	(yes/no)	0.819	0.301–2.230	0.696
ACCI	(> 5/ ≤ 5)	2.880	1.372–6.046	0.005
ASA	(≥ 3/ < 3)	4.251	1.902–9.502	< 0.001
Distance from anastomosis to anal margin	(by 1 cm)	0.825	0.656–1.038	0.101
T3	(yes/no)	1.106	0.560–2.187	0.771
T4	(yes/no)	3.014	1.270–7.152	0.012
LNM	(yes/no)	3.748	1.749–8.032	0.001
Distant metastasis	(yes/no)	3.217	1.144–9.045	0.027
Tumor type	(high/moderate/low/other)	1.168	0.760–1.795	0.480
Pathological stage	(0/1/2/3/4)	0.481	0.410–0.565	< 0.001

*BMI* Body mass index, *PNI* Prognostic nutritional index, *NLR* Neutrophil-to-Lymphocyte Ratio, *CRM* circumferential resection margin, *EMVI* Extramural venous invasion, *ACCI* Age-adjusted Charlson Comorbidity Index, *ASA* American Society of Anesthesiologists, *LNM* Lymph node metastasis

After conducting collinearity analysis, it was observed that all candidates in the univariate analysis exhibited a variance inflation factor of less than 2. Multivariate logistic regression was conducted on latent preoperative variables identified in previous univariate analyses to determine the independent risk factors associated with PS.

**Fig. 2:**
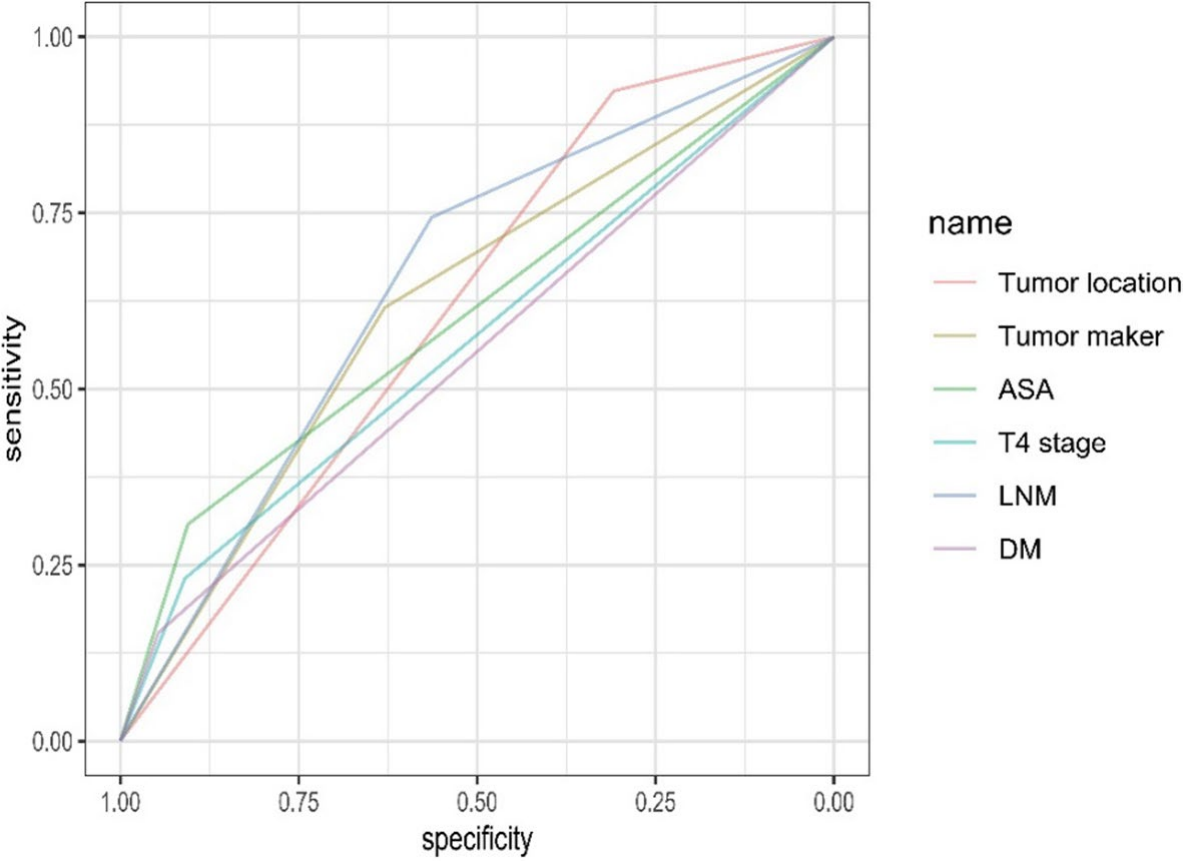
The ROC results for all independent risk factor

Multivariate logistic analysis revealed that tumor distance ≤ 6 cm (OR = 6.631, 95% CI = 1.792–24.543, *P* = 0.005), abnormal tumor markers (OR = 2.309, 95% CI = 1.059–5.031, *P* = 0.035), ASA score ≥ 3 (OR = 4.784, 95% CI = 1.626–14.070, *P* = 0.004), T4 stage (OR = 2.880, 95% CI = 1.069–7.758, *P* = 0.036), lymph node metastasis (OR = 4.566, 95% CI = 1.915–10.884, *P* = 0.001), and distant metastasis (OR = 4.478, 95% CI = 1.102–18.201, *P* = 0.036) were significantly related to a permanent stoma (Table 3). The ROC curves for each independent risk factor are depicted in Fig. 2. The AUCs of these factors were also calculated and were as follows: tumor distance (0.6159), abnormal tumor markers (0.6225), ASA score (0.6065), T4 stage (0.5701), lymph node metastasis (0.6537), and distant metastasis (0.5502).

**Table 3:** Multivariate logistic regression analysis of the notable factors

Characteristics	OR	95% CI	*P* value
Age	1.859	0.628—5.499	0.263
Tumor distance	6.631	1.792—24.543	0.005
Tumor marker	2.309	1.059—5.031	0.035
ACCI	0.838	0.279—2.515	0.752
ASA	4.784	1.626—14.070	0.004
T4 stage	2.88	1.069—7.758	0.036
LNM	4.566	1.915—10.884	0.001
Distant metastasis	4.478	1.102—18.201	0.036

Tumor location, the distance from tumor to anal margin; *ACCI* Age-adjusted Charlson Comorbidity Index, *ASA* American Society of Anesthesiologists, *LNM* Lymph node metastasis, *DM* Distant metastasis

The Omnibus test of the model coefficient indicated that the χ2 value was 54.410, *P* < 0.001, suggesting statistical significance for the logistic model. The Hosmer‒Lemeshow test indicated that the *P* value was 0.682, indicating that the model demonstrates a strong fit with the observed data.

**Fig. 3:**
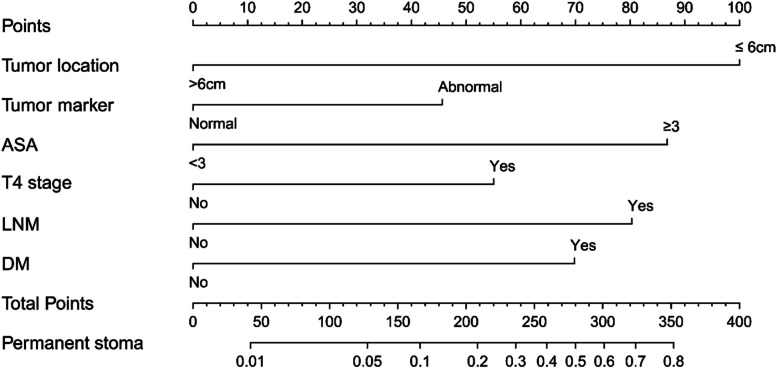
Nomogram for predicting permanent stoma after low anterior resection for rectal cancer. Tumor location, the distance from tumor to anal margin; LNM, lymph node metastasis; DM, Distant metastasis

A nomogram model was developed using the six identified independent risk factors identified in the development group to predict the probability of PS in patients with rectal cancer undergoing ileostomy (Fig. 3). The corresponding score for each factor was obtained according to the patient’s individual data, and the scores of the six factors were aggregated to derive the total score. The final predicted risk of a PS was the probability corresponding to the total score of an individual patient.

**Fig. 4:**
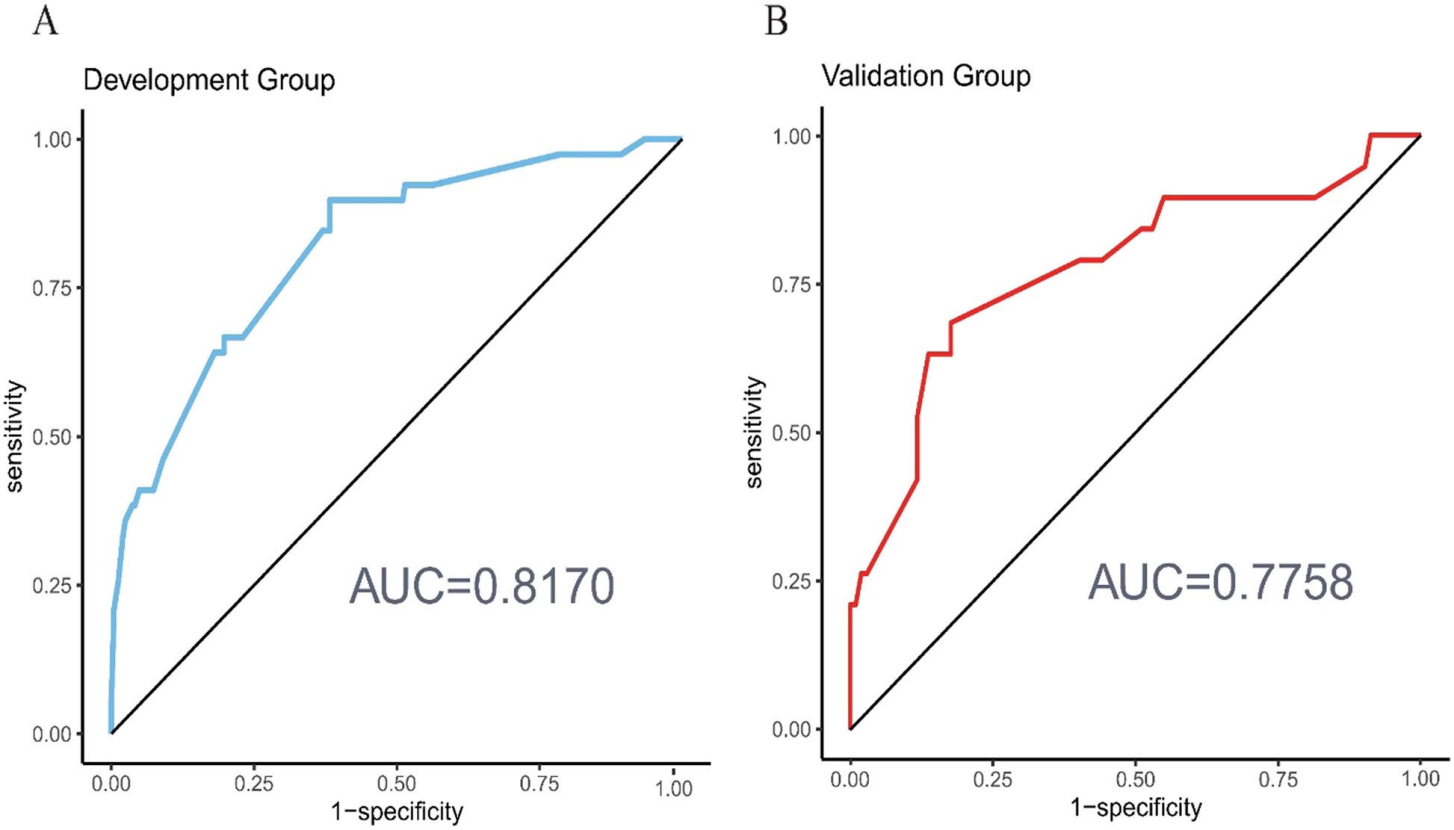
The nomogram model predicts the receiver operating characteristic ROC curve for major complications after rectal cancer surgery. AUC, area under the curve

Using an ROC curve for assessing the nomogram’s prediction accuracy, we found that the area under the ROC curve was 0.8170 (95% CI: 0.7441–0.8899) for the development set and 0.7758 (95% CI: 0.6450–0.9066) for the validation set (Fig. 4).

**Fig. 5:**
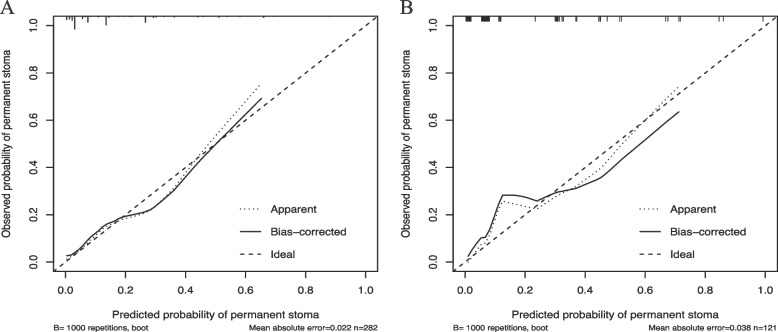
Calibration of the nomogram for permanent stoma in the development group (**A**) and the validation group (**B**). The x-axis shows the predicted probability of permanent stoma, and the y-axis shows the observed probability of permanent stoma

The calibration curves were used to assess the concordance between the predictions and observations in this study (Fig. 5). The vertical axis in the calibration curves represents the probability of actual permanent stoma occurrence, while the horizontal axis indicates the predicted probability of permanent stoma occurrence. The ideal model is depicted by a diagonal dotted line, symbolizing a flawless prediction. On the contrary, the solid line represents performance, with a stronger resemblance to the diagonal dotted line indicating enhanced predictive capability. The calibration performed on the development (a) and validation groups (b).

## Discussion

In this study, the prevalence of PS (14.4%) aligns with the prevalence range of 6–23.2% reported in the literature [12–15].

A distance from the tumor to the anal margin less than 6 cm, abnormal tumor markers, an ASA score ≥ 3, T4 stage, lymph node metastasis, and distant metastasis were identified as independent risk factors for PS after rectal cancer surgery in the multivariate logistic regression analysis. Based on these six factors, a preoperative nomogram was constructed to predict the likelihood of conversion to PS within 1 year after colorectal resection for TS patients.

Among the parameters of this model, tumor distance was a high-risk factor for a PS, which may be related to the occurrence of anastomotic leakage after surgery for low rectal cancer [16, 17]. In the 1990s, Rullier et al. reported that anastomoses located less than 5 cm from the anal verge had a risk of leakage that was 6.5 times higher, and Vignali et al. reported a seven-fold increased risk of leakage following stapling in low rectal cases [18, 19]. In a 2022 study involving 13,175 patients, the proximity of the tumor to the anal margin was identified as a significant risk factor for anastomotic leakage [20].

Anastomotic leakage after rectal cancer resection often results in a permanent stoma [21]. In a study by Kang et al., the distance of the tumor from the anal verge was indicative of the likelihood of permanent stoma formation, which was consistent with our findings [22]. However, a separate study revealed that tumor location did not demonstrate independent significance as a risk factor for PS, which may differ from the cutoff point of tumor distance across studies [23]. Additionally, surgeons have different understandings about whether patients need ileostomy, which leads to some patients with low rectal cancer not undergoing ileostomy during surgery.

This may be related to the different truncation values for tumor locations across studies.

We found that patients whose preoperative tumor marker levels exceeded normal values were more likely to keep a PS. Tumor markers, such as CEA, CA 19–9, CA 125, and AFP, are cell surface glycoproteins produced by cancer cells and indicate the malignant characteristics of tumors [24–26].

Granell et al. studied preoperative CEA levels and p53 expression in 134 patients with colorectal cancer and found that patients with elevated preoperative CEA levels had a significantly higher risk of local recurrence within 2 years [27]. Other studies have also shown that the presence of elevated preoperative CEA levels is significantly associated with advanced or metastatic disease, indicating a more unfavorable prognosis [28]. Commonly used serum tumor markers are closely associated with the prognosis of patients with colorectal cancer [29–31]. Abnormal tumor marker levels suggest a poor prognosis and are correlated with a PS.

Multiple previous reports have shown that an ASA score ≥ 3 is an independent risk factor for a PS [32, 33]. In this study, patients with ASA scores ≥ 3 were 4.78 times more likely to have a PS than those with lower scores, suggesting that they may be more prone to postoperative complications, which may make them less likely to have a second procedure recommended due to contraindications or even refuse the reversal because of fear. As mentioned previously, a higher ASA score is associated with a higher risk of anastomotic leakage [34, 35].

Therefore, for such patients, surgeons should strengthen communication before the operation to avoid unnecessary conflicts between doctors and patients.

Simultaneously, we observed an increased risk of PS in cancer patients with T4 stage disease and metastatic lymph nodes, which can be attributed to the increased technical difficulty of surgical procedures in such patients [36]. Wang et al. found that T3-4 stage was an independent risk factor for anastomotic leakage [37]. Moreover, the probabilities of recurrence and metastasis increase in patients with locally advanced disease. In clinical practice, patients with distant metastasis face a bleak prognosis and reduced life expectancy, accompanied by an increased likelihood of developing mechanical ileus due to tumor recurrence and peritoneal seeding. These risks have a significant impact on intestinal tract closure. Furthermore, these patients often require an increased number of chemotherapy cycles, and the presence of ileostomy-associated renal dysfunction as well as imbalances in water and electrolyte levels may compromise their ability to tolerate chemotherapy, whereas chemotherapy-induced liver and kidney function impairment and myelosuppression may delay the second procedure.

Our nomogram provides surgeons with the probability of a PS in patients undergoing rectal resection with ileostomy and can be fully utilized prior to deciding on the type of surgical procedure. When patients with a high probability of a PS are identified using the nomogram, it is necessary to enhance preoperative communication with patients and their families so that they can fully understand the possibility of a PS postoperatively and the corresponding complications. Closure of the temporary stoma may not always be possible, and a Hartmann procedure or establishment of a colostomy may be better options.

In this study, we found that the postoperative pathological stage was also related to keeping a PS, which is expected given that a higher preoperative stage is a risk factor for a PS.

The choice between ileostomy and colostomy presents a persistent dilemma for patients diagnosed with low rectal cancer. A meta-analysis of 1529 patients showed that ileostomy was associated with a lower risk of ostomy [38]. Another study involving 1687 patients also found that temporary ileostomy had less impact on patients and recommended temporary ileostomy instead of colostomy after low anterior resection for rectal cancer [39]. Moreover, a study involving 2036 patients demonstrated that ileostomy has a higher incidence of complications than colostomy [10]. However, it has been noted in various studies that each type of dysfunctional ostomy possesses its own set of advantages and disadvantages, and there is currently insufficient evidence to support the superiority of one temporary ostomy over colorectal anastomosis [40, 41, 42].

A slight preference exists for performing ileostomy in patients with low rectal cancer. However, it is worth noting that the primary objective of our study was to identify patients who are unsuitable for temporary ileostomy placement, and not all patients should choose colostomy. These two types of stoma possess distinct advantages and disadvantages in different scenarios, necessitating a meticulous selection process, which constitutes the primary objective of our study. One of the key advantages of our study lies in the utilization of this nomogram, which enables us to preoperatively identify patients with a higher likelihood of having a PS. Our findings can provide valuable insights for enhancing surgical practice. A key point for clinicians to consider is the importance of communication with the patient and family and a realistic expectation of when or if a stoma will be reversed. During the telephone follow-up, we noticed that in addition to clinical factors, the patient's subjective will and the family's economic situation also accounted for a relatively important part of keeping a PS, which was a deficiency in our study. Another potential limitation of the study is that factors relevant to the surgical process were not considered in the model. To improve this research to the level of machine learning and achieve more accurate results, various algorithms, such as decision trees, random forests, support vector machines, and XGBoost, will be employed for cross-validation of the development group. This direction also aligns with our future research goals. In addition, as this was a single-center study and was not validated using external data, a prospective or multicenter study should be conducted to better validate the sensitivity and specificity of our nomogram.

## Conclusion

This study analyzed the preoperative risk factors for a PS and developed a predictive model that can accurately differentiate patients who will keep a PS. Those with a high probability of developing a PS may consider colostomy for better quality of life and outcomes.

## Data Availability

The datasets used and/or analyzed during the current study are available from the corresponding author on reasonable request.

## Abbreviations

TI: Temporary Ileostomy
PS: Permanent Stoma
TS: Temporary Stoma
AUC: Area under the Curve
ROC: Receiver Operating Characteristic
ACCI: Age-adjusted Charlson Comorbidity Index
ASA: American Society of Anesthesiologists
LNM: Lymph Node Metastasis
DM: Distant Metastasis
